# White spongy nevus in a young patient: Diagnostic challenges and clinical insights

**DOI:** 10.6026/973206300222742

**Published:** 2026-05-31

**Authors:** Sailaja Choudary A, Sheethal H.S, Kavitha Rao, Abhinethra M.S, Manjula N., Manisha Rao, Meenal Kate

**Affiliations:** 1 Department of Oral Medicine and Radiology, Vokkaligara Sangha Dental College and Hospital, Bangalore, India; 2 Department of Oral Maxillofacial Pathology, Vokkaligara Sangha Dental College and Hospital, Bangalore, India; 3 Department of Prosthodontics, Vokkaligara Sangha Dental College and Hospital, Bangalore, India; 4 Department of Oral Medicine, Vokkaligara Sangha Dental College and Hospital, Bangalore, India; 5 Department of Oral Pathology, Vokkaligara Sangha Dental College and Hospital, Bangalore, India

**Keywords:** White spongy nevus (WSN), hereditary mucosal disorder, buccal mucosa, defect of keratinization, differential diagnosis, histopathology, oral white lesion, case report

## Abstract

White spongy nevus (WSN) is an infrequent hereditary mucosal disorder that is asymptomatic and is marked by white corrugated plaques due to defects in epithelial keratinization. Therefore, it is of interest to report of a 20-year-old woman who has bilateral mucosal lesions of the buccal area that are non-responsive to antifungal treatment. WSN was established through clinical features and histopathology which include hyperparakeratosis, vacuolated spinous cells and perinuclear eosinophilic condensation. WSN is a benign disorder; however, it is often confused and should be distinguished with malignant and infectious lesions. Its clinical and genetic background is important for counselling and follow-up

## Background:

White spongy nevus (WSN) is a rare, benign hereditary disorder that primarily affects the mucous membranes, most commonly the oral cavity. It is characterized by the presence of asymptomatic white plaques affecting the oral mucosa and, less frequently, the esophageal and genital mucosa. The condition was first described by Hyde in 1909 and has been referred to by several names, including Cannon's disease, hereditary leukokeratosis of the mucosa and white spongy nevus of Cannon [1]. WSN is associated with genetic mutations that lead to defective keratinization. These mutations typically affect keratin genes responsible for maintaining the structural integrity of epithelial cells, resulting in abnormal keratinization within non-keratinized stratified squamous epithelium. The altered keratin production leads to the characteristic spongy, folded appearance of the mucosal lesions [2]. Clinically, the lesions appear as diffuse, thickened white plaques with a folded or corrugated surface. They are usually asymptomatic and do not interfere with normal oral functions such as speech, mastication, or swallowing. Because of their benign nature, these lesions are often discovered incidentally during routine oral examination. Therefore, it is of interest to report describes a female patient presenting with characteristic lesions affecting both the right and left buccal mucosa.

## Case description:

A Fathima 20 year's patient presented to the department with a case of difficulty in biting because of her FPD left lower back teeth area. The past history of illness revealed that she has just received her prosthesis 10 days ago. She also moaned of patch on her cheeks, which she was offered topical clotrimazole by her dentist with no effects. No such patch had been had among family history in the past by her siblings. During the questionnaire regarding her father and mother family members, she disclosed that her uncle had a daughter who had had the same patches in her mouth. General examination with all the vital signs showed normal values. Systemic assessment and extra oral examination did not show any abnormality. Intraorally, there is a white translucent corrugated patch on the bilateral buccal mucosa extensively on right buccal mucosa (almost 3* 2.5 cm) and on right side (almost 1.5*2 cm). They were non scrapable although there was mild sloughing. The rest of the oral mucosa did not have any similar lesions. Palpation revealed that it was non tender, smooth and, on stretching of the mucosa, it remains the same. Topical clotrimazole did not indicate any changes; hence we recommended biopsy with possibility of white spongy nevus. A provisional diagnosis of white spongy nevus was made and a differential diagnosis of pseudomembranous candidiasis, verrucous leukoplakia and dyskeratosis congenita was made based on history and clinical examination. Other haematological tests were carried out with values that were within normal limits, thus we used incisional biopsy as investigative procedure. Histopathy disclosed hyperplastic squamous epithelium in the form of broad and bulbous rete ridges. There was hyper parakeratosis. There were cytoplasmic vacuolization and pyknotic nuclei detected in superficial spinous layer. There was perinuclear eosinophilic condensation. Focal areas were dyskeratotic and mildly basilar hyperplastic. There was a moderate connective tissue underneath with diffuse moderate inflammatory infiltrate which was mostly lymphocytes. Many budding capillaries and blood vessels which were lined by endothelium were observed. Candidal hyphae were not seen on PAS stained sections. Based on the clinical and histopathological findings, the disease was diagnosed with White Sponge Nevus of buccal mucosa (bilateral). As white spongy nevus is a benign lesion with no treatment necessary. We had prescribed her chlorhexidine mouth wash and recommended removal of lesion due to cosmetic reasons. We had requested her to notice whether there were any changes and check and monitor.

## Discussion:

White spongy nevus (WSN) is considered a rare hereditary mucosal disorder that is primarily diagnosed based on its clinical presentation and a positive family history. In more disseminated or atypical cases, histopathological and genetic analyses are often required to confirm the diagnosis. The condition typically presents as a non-reactive white lesion that appears at birth or during early childhood, making early onset an important diagnostic feature [1], [2]. In the differential diagnosis of persistent white oral lesions, several conditions must be considered. A history of tobacco use is commonly associated with leukoplakia, which may clinically resemble WSN. Non-homogeneous leukoplakia often presents with a corrugated surface and multiple keratotic plaques with rough surface projections. Importantly, leukoplakia is classified as an oral potentially malignant disorder with a reported malignant transformation rate ranging from approximately 4% to 47%, necessitating careful monitoring and long-term follow-up [3]. Another condition that may present with white oral lesions is chronic hyperplastic candidiasis caused by Candida albicans. In such cases, lesions may be associated with local trauma, systemic disease and erythematous areas that become visible after partial removal of the lesion. Unlike WSN, chronic candidiasis generally lacks a familial pattern and histopathological examination reveals fungal spores and pseudohyphae within the epithelium [4]. Pachyonychia congenita can also mimic white spongy nevus because both conditions present with keratotic white mucosal lesions. However, pachyonychia congenita is typically associated with additional systemic features, particularly nail dystrophy and characteristic skin lesions, which help distinguish it clinically from WSN [5]. Oral lichen planus may also be considered in the differential diagnosis. This condition is frequently associated with systemic diseases such as diabetes mellitus, hypothyroidism and hepatitis C infection. Unlike WSN, oral lichen planus commonly exhibits the characteristic Wickham's striae pattern and generally occurs in adults, whereas WSN is more commonly observed in patients younger than 30 years of age [6], [7]. In some reported cases, lesions initially considered benign have demonstrated progression to moderate or severe epithelial dysplasia during follow-up periods extending up to one year. Therefore, genetic mutations, lifestyle risk factors such as smoking and alcohol consumption and a family history of carcinoma should be carefully evaluated when assessing patients presenting with persistent white oral lesions [8], [9], [10]. At present, the effectiveness of available therapeutic approaches for WSN remains limited. Various treatment modalities have been attempted; however, many reports describe minimal or inconsistent clinical improvement [11], [12]. The epithelial folds characteristic of WSN are believed to create an environment that may support the growth of non-pathogenic microorganisms. This phenomenon has been suggested as a possible explanation for the occasional improvement observed with antibiotic therapy in some reported cases [13], [14], [15].

## Conclusion:

WSN is a rare condition to identify and prevent possible confusion with serious lesions. The knowledge of clinical genetic origin guarantees correct diagnosis and minimises unwarranted intervention. Moreover, follow up of patients over a long period to study the progression of the disease and the malignant transformation is necessary.

## Funding:

Nil

## Declaration:

The case has not been presented in any meeting or conference. The identity of the patient is been strictly confidential and the consent of the patient has been taken with due respect and consideration.
